# Graphene nanoplatelets based matrix solid-phase dispersion microextraction for phenolic acids by ultrahigh performance liquid chromatography with electrochemical detection

**DOI:** 10.1038/s41598-017-07840-2

**Published:** 2017-08-08

**Authors:** Li-Qing Peng, Ling Yi, Qiu-Cheng Yang, Jun Cao, Li-Jing Du, Qi-Dong Zhang

**Affiliations:** 1 College of Material Chemistry and Chemical Engineering, Hangzhou Normal University, Hangzhou, 310036 China; 2 Drug Clinical Trial Institution, The First Affiliated Hospital of Soochow University, Suzhou, 215006 P. R. China

## Abstract

A simple, rapid and eco-friendly approach based on matrix solid-phase dispersion microextraction (MSPDM) followed by ultrahigh performance liquid chromatography coupled with electrochemical detection (UHPLC-ECD) was presented for the microextraction and determination of six phenolic acids in a plant preparation (Danshen tablets). The parameters that influenced the extraction performance of phenolic acids were investigated and optimized. The optimal MSPDM conditions were determined as follows: sorbent, using graphene nanoplatelets with sample/sorbent ratio of 1:1, grinding time set at 60 s, and 0.2 mL of water as elution solvent. Under the optimum conditions, the validation experiments indicated that the proposed method exhibited good linearity (r^2^ ≥ 0.9991), excellent precision (RSD ≤ 4.57%), and satisfactory recoveries (82.34–98.34%). The limits of detection were from 1.19 to 4.62 ng/mL for six phenolic acids. Compared with other reported methods, this proposal required less sample, solvent and extraction time. Consequently, the proposed method was successfully used to the extraction and determination of phenolic acids in Danshen tablets.

## Introduction

Graphene, the two-dimensional carbon nanomaterial composed of monolayered sp^2^-hybridized carbon atoms and arranged in a honeycomb network, has attracted tremendous attention in recent years due to its characteristics of high elasticity, chemical stability, excellent mechanical strength, large specific surface area (2630 m^2^/g), high electrical and thermal conductivity, large delocalized π-electron system, biocompatibility, and strong Young’s modulus^1–7^. Graphene has been used as adsorption material and electrochemical sensors due to its ultrahigh specific surface area and π-π electrostatic stacking property which can be used to adsorb benzene ring compounds^8^. In addition, the graphene and some graphene-based composites are applied in various fields of nanoelectronic devices, drug delivery, electronics, chemical and biosensors, energy storage, and catalysis^9–12^. However, it is very hard and complex to obtain monolayer graphene from graphite, therefore, the graphene nanoplatelets (GnPs) with several layers of graphene are extensively studied owing to its easier preparation^13^. GnPs with an average thickness about 5–25 nanometers and a diameter in the range of 0.5 to 25 μm are stacked by 10–30 graphene sheets^14^ and yet retain the single layer properties^15^. The GnPs can be used in many applications such as loading catalysts^16^, sensing applications^17^ and adsorbent materia^18^. In particular, as far as we know, there is no report about the use of GnPs in the microextraction process.

Microextraction methods, which arised from the evolution of traditional sample pretreatment towards simplicity and miniaturization to extract various organic and inorganic analytes, can be categorized in two general classes: liquid-phase microextraction (LPME) and solid-phase microextraction (SPME). Compared with classical extraction methods such as liquid-liquid extraction (LLE) and solid-phase extraction (SPE), the microextraction methods have lots of advantages, such as low consumption of solvent and sample, generating less wastes, giving high enrichment factors and requiring less extraction time^19^. Recently, matrix solid-phase dispersion (MSPD), another extraction method with advantages of simplicity and high enrichment factor has attracted increasing attention. MSPD, introduced by Barker in 1989^20^, is a simple and cheap sample preparation method which integrates sample homogenization, disruption, extraction, fractionation and purification in one step^21^. The MSPD method involves blending and grinding a solid, semisolid or viscous sample with a suitable solid adsorbent to form an apparent homogeneous mixture and isolating target compounds by desorption with a small amount of elution solvent indicating the unique properties of simple preparation, short analytical time, exclusion of sample component degradation, high extraction efficiency and the reduction of sample and organic solvent consumption^22, 23^. Nowadays, the MSPD method has been widely applied to analysis of target compounds in various samples, such as food, plant, biological and environmental samples^24^. Nevertheless, the traditional MSPD methods require relatively large amount of sample, adsorbent and organic solvent^25^, a novel and environmentally friendly MSPD microextraction (MSPDM) method^26, 27^, therefore, has developed and attracted more and more attention for the saving of time, sample and solvent. The choice of a suitable sorbent material is a significant factor in MSPDM process. Commonly used adsorbents are silica gel, C_18_ and florisil, but they show low selectivity for analytes and low efficiency in the extraction of the low content compounds^28^. Hence, it is important to seek the more selective and efficient adsorbents. GnPs, with its exceptional features, could be a potential great sorbent, and yet there is no report on the application of GnPs as the sorbent in MSPDM so far to our best knowledge.

The dried root of *Salvia Miltiorrhiza* Bunge (Danshen) is a commonly used herbal medicine which was applied to the therapy of numerous ailments such as chronic nephritis, arthrophlogosis, coronary heart diseases, cardiovascular disease, cerebrovascular diseases, hepatitis, hypertension, and neurasthenic insomnia^29–32^. In recent years, multiple Danshen preparations such as Fufang Danshen Dripping Pill, compound Danshen tablets, Danshen tablets, Danshen capsules and Danshen injections have been used in relieving pain, eliminating blood stasis, promoting blood flow and the treatment of cardiovascular problems^33^. Danshen preparations are rich in water-soluble phenolic acids including sodium danshensu, rosmarinic acid, protocatechuic aldehyde, protocatechuic acid, lithospermic acid, salvianolic acid B, salvianolic acid A and other minor constituents^34^. The phenolic acids are the main biological active components which demonstrate pharmacological effects including antioxidant, anti-apoptosis and vasodilation^30, 35^. As a result, the extraction and determination of phenolic acids from Danshen and its preparations are very significant for evaluation of their clinical efficacy and safety. According to literature reports, several pretreatment methods for isolation and preconcentration of active compounds from Danshen and its preparations were used, including ultrasound assisted extraction^36^, ionic liquid-based ultrahigh pressure extraction^37^, shake and sonication extraction^38^, solid-phase extraction^39^, sonication^40^ and ultrasound-assisted ionic liquid-based homogeneous liquid-liquid microextraction^41^ prior to chromatography analysis notwithstanding the labor cost, high consumption of organic solvent and extraction time. Therefore, it is important to develop a miniaturized environmentally friendly extraction method.

The aim of this study is to establish a simple, sensitive, rapid and environmentally friendly MSPDM method coupled with ultrahigh performance liquid chromatography coupled with electrochemical detection (UHPLC-ECD) for the simultaneously extraction and determination of phenolic acids in plant preparation (Danshen tablets). After optimizing the potential influencing parameters, like type of sorbent, sample/sorbent ratio, the grinding time, and the type of elution solvent, this method is validated in terms of linearity, limits of detection and quantification, precision and accuracy, and then successfully applied to the extraction and determination of phenolic acids in Danshen tablets, a case in point of plant preparation.

## Results and Discussion

### Characterization of material

**Figure 1:**
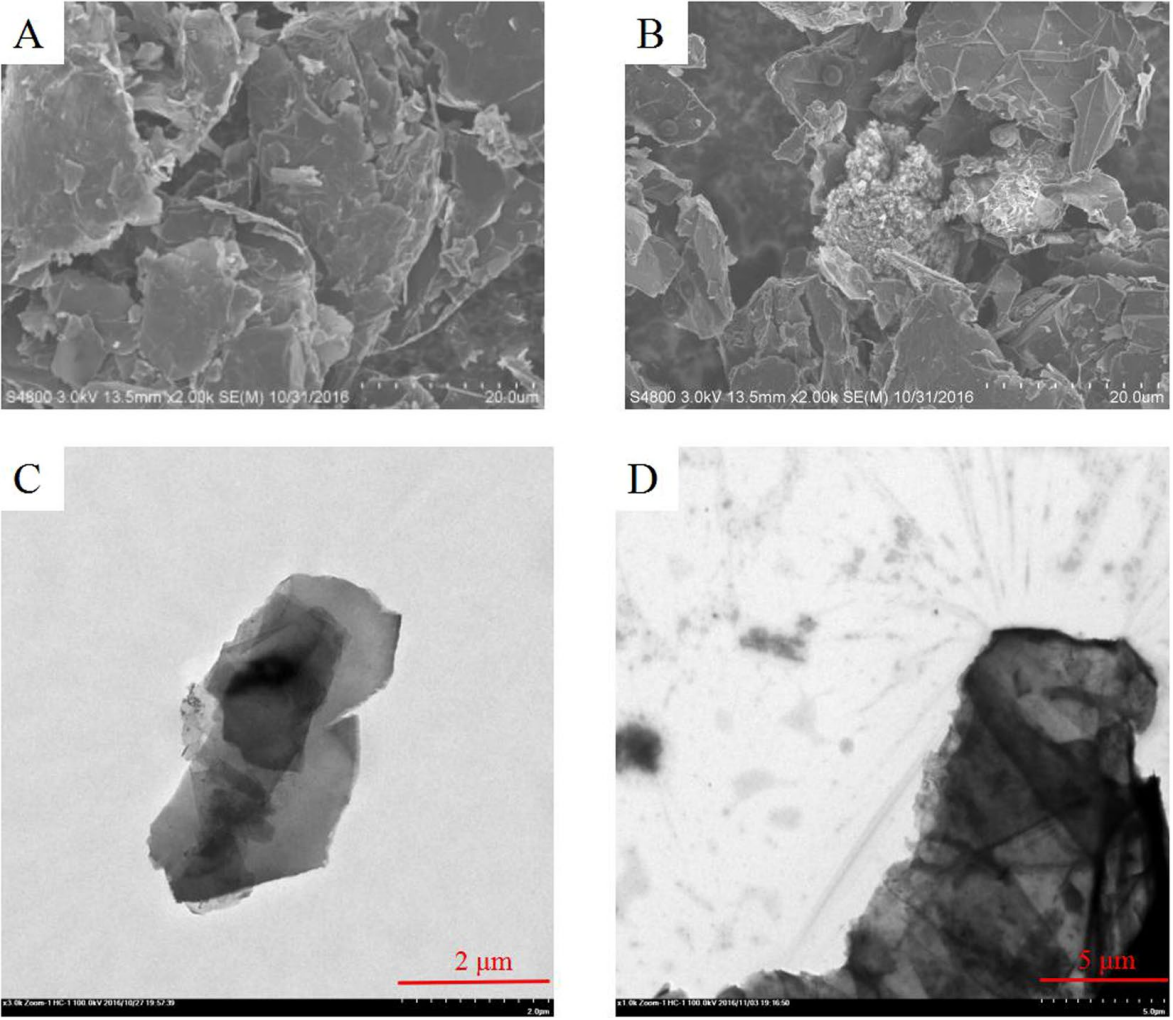
SEM and TEM images of GnPs and GnPs-Danshen tablets, (**A**) and (**B**) show the SEM images of GnPs and GnPs-Danshen tablets, respectively, (**C**) and (**D**) show the TEM images of GnPs and GnPs-Danshen tablets, respectively.

**Figure 2:**
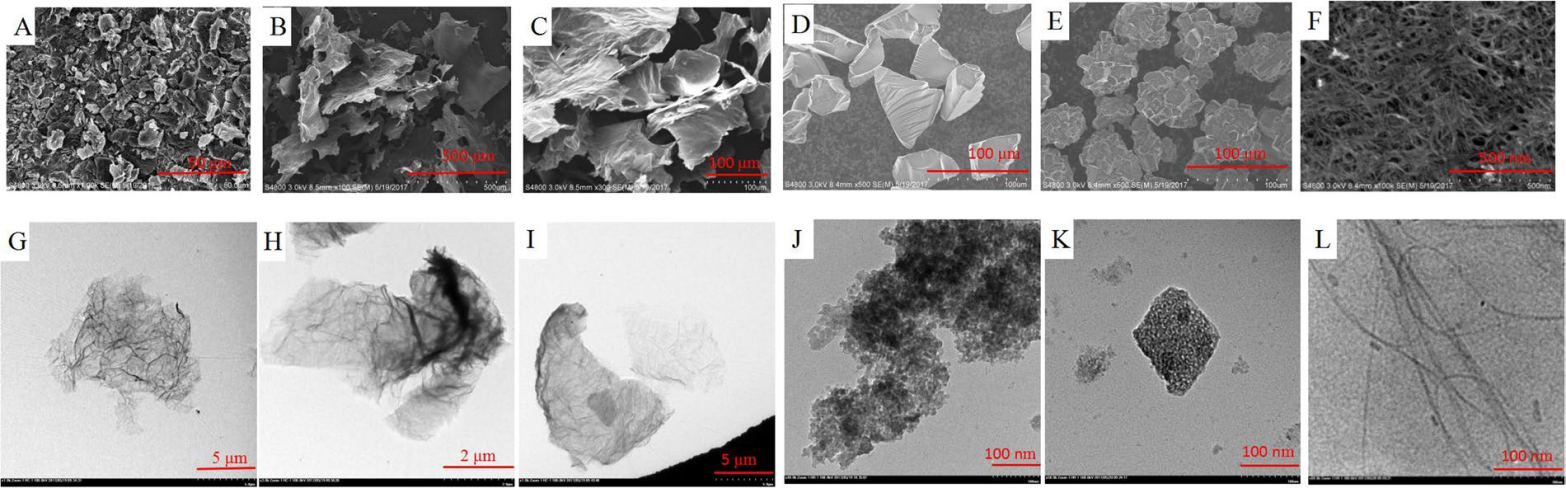
SEM and TEM images of graphene (**A**,**G**), graphene oxide (powder) (**B**,**H**), graphene oxide (sheet) (**C**,**I**), Al_2_O_3_ (**D**,**J**), C18 (**E**,**K**) and SWNT (**F**,**L**).

The scanning electron microscope (SEM) images of GnPs and GnPs-Danshen tablets displayed in Fig. 1A,B were used to observe the morphology of GnPs and the GnPs grinded with Danshen tablets. As seen from the Fig. 1A, the GnPs have a platelike structure and several graphene layers were stacked together to form the clusters. Figure 1B showed that the Danshen tablets were adsorbed to the GnPs, and the surfaces of Danshen sample were rough with some gaps indicating the active components are extracted by the GnPs. The transmission electron microscopy (TEM) images of GnPs and GnPs-Danshen tablets are presented in Fig. 1C,D in order to acquire the detailed microstructures of the GnPs before and after grinded with the Danshen tablets. The TEM image of GnPs (Fig. 1C) demonstrated that the GnPs were in translucent lamellar structure (~5–7 μm diameter) which had some wrinkle and embossment on the surface. The black area indicated the overlapping region while the gray area indicated a thinner area. Compared with the TEM of GnPs. Figure 1D exhibited some black substances (Danshen tablets) on the edge and surface of the GnPs indicating the existence of the interaction between the GnPs and Danshen tablets which resulted in a good adsorption for the samples. In addition, the SEM and TEM images of graphene, graphene oxide (powder), graphene oxide (sheet), Al_2_O_3_, C18 and single-walled carbon nanotube (SWNT) were displayed in Fig. 2. The graphene (Fig. 2A,G) with a diameter of about 11–12 μm, graphene oxide (powder) (Fig. 2B,H) with a diameter of about 7–8 μm and graphene oxide (sheet) (Fig. 2C,I) with a diameter of about 12–13 μm all showed a much thinner and more transparent laminar structure than the GnPs. Besides, the graphene oxide exhibited some distortions and defects possibly caused by the introducing oxygen groups. C18 nanoparticles with the average particle size of about 10 nm in diameter (Fig. 2J) were accumulated together to form irregular clumps in diameters of about 50–80 μm (Fig. 2D). Figure 2E showed that the Al_2_O_3_ has a agglomerated morphology with a featured size around 50 μm formed by lumps of nanoparticles about 150–200 nm in diameter (Fig. 2K). The SWNT had a diameter of about 2–3 nm (Fig. 2L) and they agglomerated together as seen from Fig. 2F.

**Figure 3:**
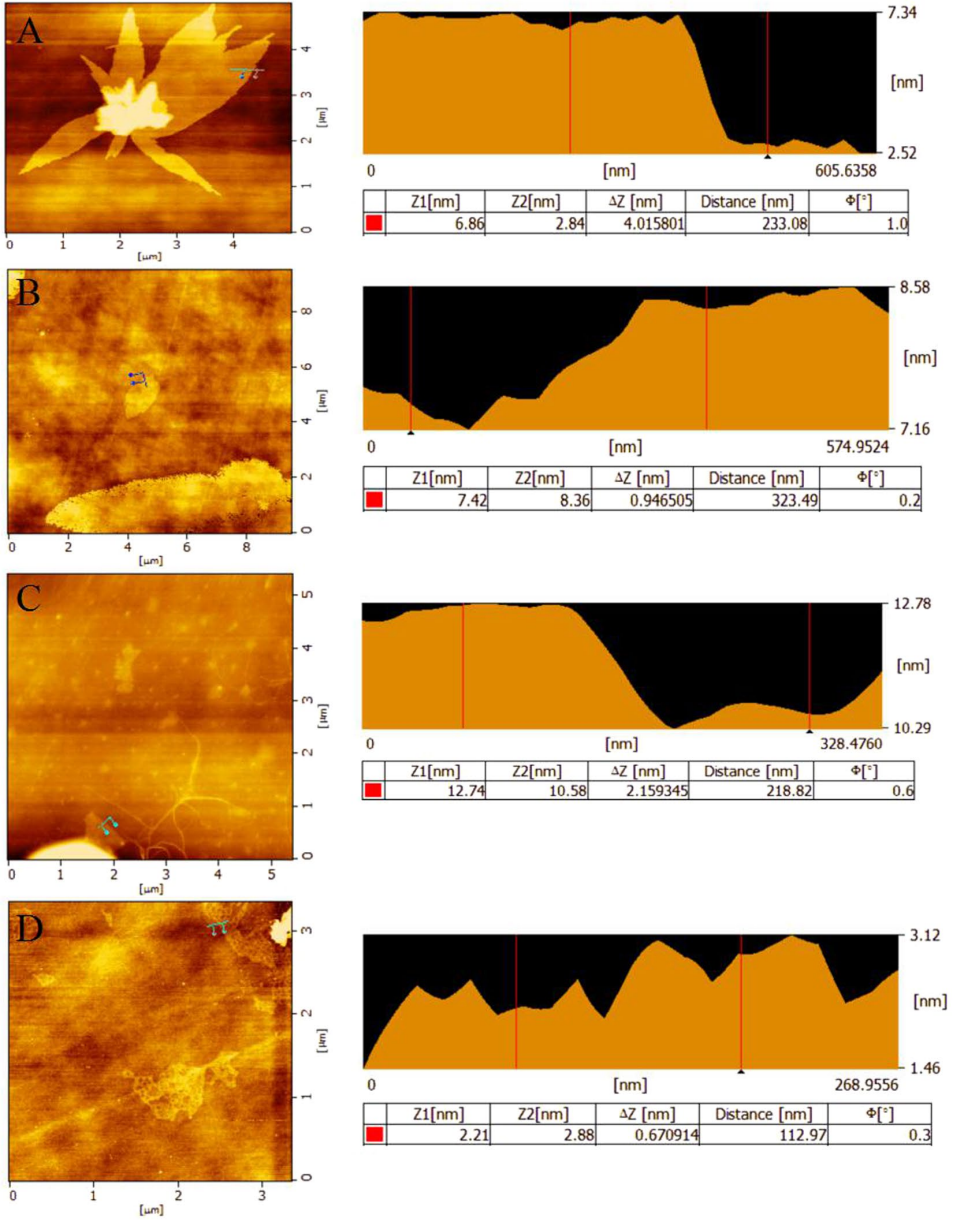
AFM images of GnPs (**A**), graphene (**B**), graphene oxide (powder) (**C**) and graphene oxide (sheet) (**D**).

The surface morphology of GnPs, graphene, graphene oxide (powder) and graphene oxide (sheet) were further investigated by atomic force microscope (AFM) (Fig. 3). As is shown in the AFM images, the GnPs (Fig. 3A) possessed a thickness of ~4.02 nm indicating the presence of about 10 layers of graphene. Figure 3B showed that the graphene had a thickness of ~0.95 nm that was close to the thickness of two graphene nanosheets, which might be given rise to the overlapping of the monolayer nanoplatelets on the Si substrate during deposition. Figure 3C indicated that the graphene oxide (powder) had a thickness of ~2.16 nm that was near to the thickness of three graphene oxide nanosplatelets. The thickness of graphene oxide (sheet) showed in Fig. 3D was about 0.67 nm, implying the presence of monolayer graphene oxide nanoplatelet on the substrate.

**Figure 4:**
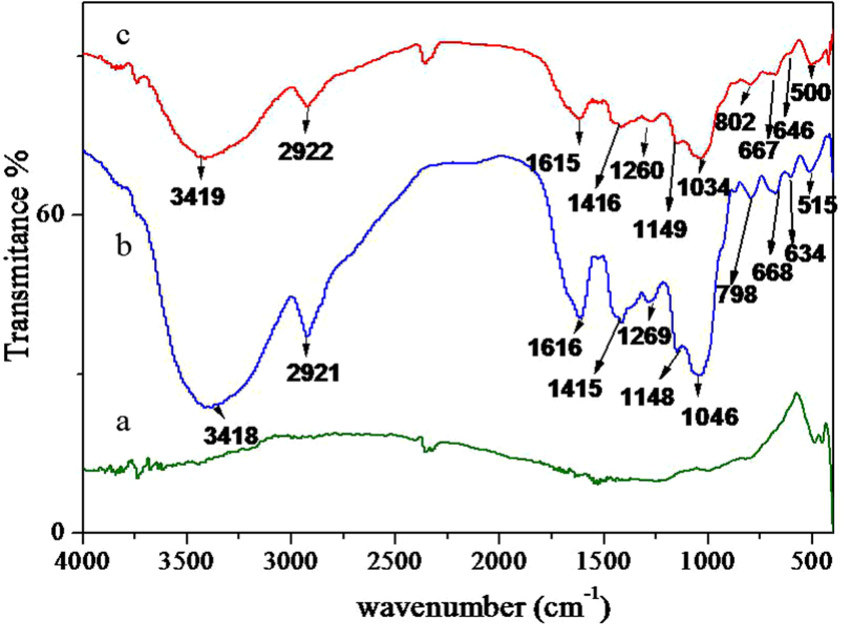
FT-IR spectra of (**a**) GnPs; (**b**) Danshen tablets; (**c**) GnPs-Danshen tablets.

The FT-IR spectrograms which provided the information of functional groups on the surface of GnPs, Danshen tablets and the GnPs-Danshen tablets were displayed in Fig. 4. It was observed that, the spectrum of GnPs showed no typical absorption features (curve a), while the IR spectrum of Danshen tablets (curve b) displayed a number of characteristic absorption peaks which represented some specific functional groups of some containing chemicals. For instance, the adsorption peak at 3418 cm^−1^ was contributed by the stretching vibration of -OH groups in phenolic acid compounds^42, 43^, the adsorption peak at 2921 cm^−1^ was assigned to the alkyl stretching vibrations of C-H in -CH_3_ and -CH_2_ groups^42, 44^, the peak at 1616 cm^−1^ belonged to the aromatic stretching vibration and the stretching vibration of C=O groups in phenolic acids^43, 44^, a peak at 1415 cm^−1^ was allocated to the corresponding bending vibration of C-H in =CH groups^43^, the band region at 1269 cm^−1^ was resulted from the bending vibration of C-CO and O-H in phenolic acids^43, 44^, the adsorption peak at 1148 cm^−1^ was ascribed to the C-C stretching vibrations and =C-H bending vibration of benzene ring in-plane deforming^44^, and, the peak at 1046 cm^−1^ were mainly attributed to the C-O-C stretching vibration and C-O bending vibrations^42, 44^. Furthermore, the IR spectrum of GnPs-Danshen tablets (curve c) exhibited similar trends to that of Danshen tablets (curve b), only a slight shift in the wave number was observed due to the formation of inter-molecular forces between GnPs and Danshen tablets. As a result, the FT-IR analyses confirmed that the Danshen tablets had been adsorbed into the GnPs successfully.

### Type of sorbent

**Figure 5:**
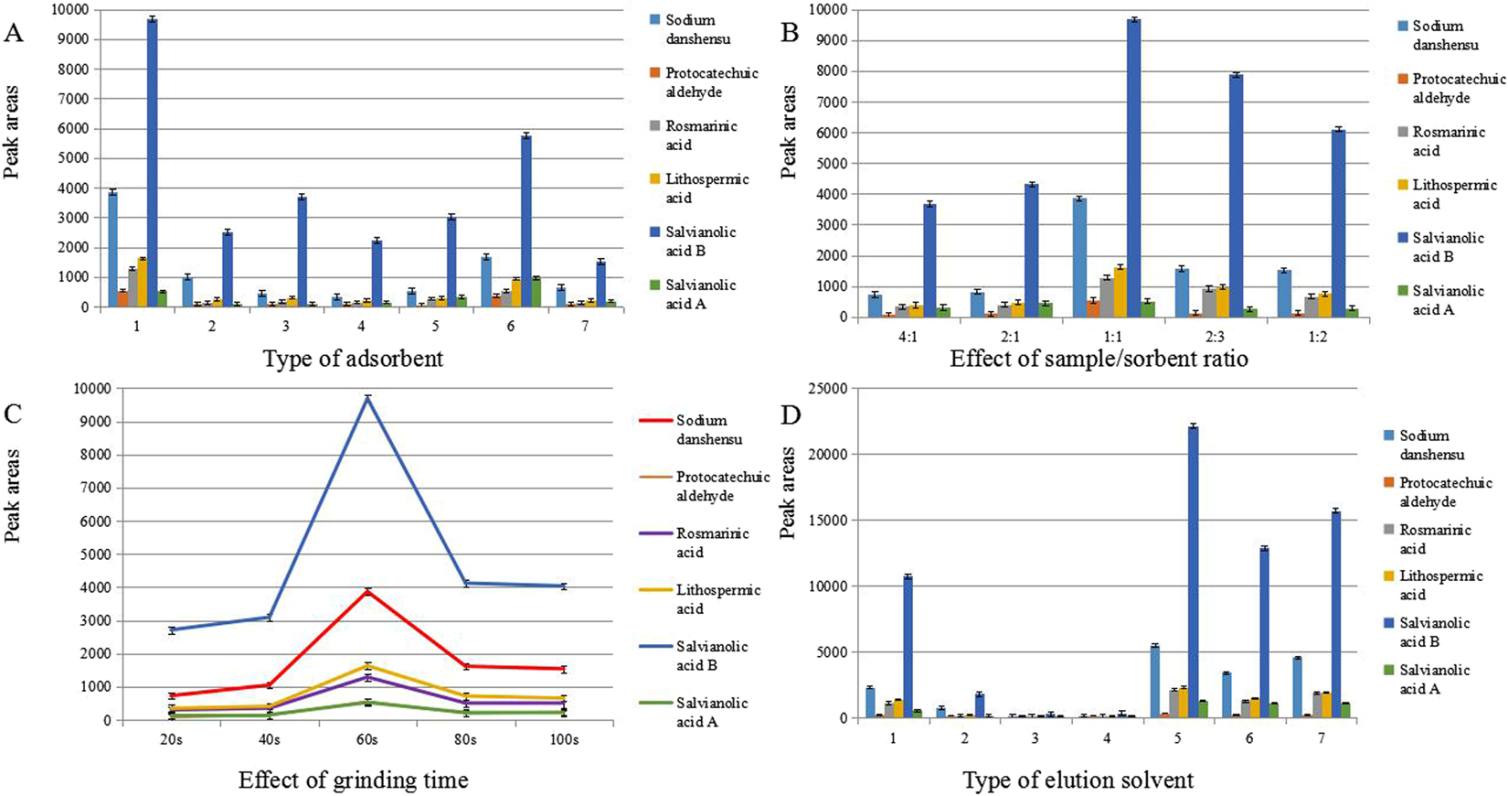
The optimization of MSPDM process. 24 mg of Danshen tablets were used and the volume of elution solvent was 0.2 mL. (**A**) Effect of the type of sorbent on the extraction efficiency of phenolic acids. Extraction conditions: amount of sorbent, 24 mg; grinding time, 60 s; type of elution solvent, methanol. Type of sorbent: (1) GnPs, (2) graphene, (3) graphene oxide (sheet), (4) graphene oxide (powder), (5) Al_2_O_3_, (6) C18, (7) SWNT. (**B**) Effect of sample/sorbent ratio: 4:1, 2:1, 1:1, 2:3 and 1:2. Extraction conditions: type of sorbent, graphene nanoplatelets; grinding time, 60 s; type of elution solvent, methanol. (**C**) Effect of grinding time: 20 s, 40 s, 60 s, 80 s, 100 s. Extraction conditions: type of sorbent, GnPs; amount of sorbent, 24 mg; type of elution solvent, methanol. (**D**) Effect of type of elution solvent: (1) methanol, (2) ethanol, (3) acetonitrile, (4) acetone, (5) water, (6) methanol-water (80:20, v/v), (7) methanol-water (60:40, v/v). Extraction conditions: type of sorbent, GnPs; amount of sorbent, 24 mg; grinding time, 60 s.

The type of sorbent plays a vital role in the MSPDM procedure, since it would not only can influence the extraction efficiencies and selectivity of target analytes in the adsorption process, but also affect the elution process. Different type of the sorbent had different physicochemical properties which could impact the extraction performance obviously. In this work, several types of sorbents including GnPs, graphene, graphene oxide (sheet), graphene oxide (powder), Al_2_O_3_, C18 and SWNT were investigated for the extraction of the investigated phenolic acids from Danshen tablet samples. As shown in Fig. 5A, the extraction performance obtained by using these sorbent displayed varied extraction yields presenting by peak areas, among which the GnPs exhibited the highest for all analytes. This may be ascribed to the fact that the GnPs possessed unique chemical structure such as the flat structure, large delocalized π-electron system, several layers of graphene sheets and large surface area which could form π-π stacking interaction, Van der Waals forces, and electrostatic interaction with the phenolic acids which had the polar groups (-OH, -COOH). Besides, the target analytes could be adsorbed into the defects and space between graphene layers of GnPs. The graphene and SWNT provided relatively poor extraction yields, possibly due to their few layers or single wall structure which would result in a poor adsorption capacity owing to the very few defects on the wall, as the defects between layers would be conducive to the adsorption of analytes. Worse still, the SWNT had smaller surface area than graphene which was unfavourable for the adsorption. Regarding the graphene oxide (sheet), graphene oxide (powder), Al_2_O_3_ and C18, they contained lots of oxygen atoms (O) which could form hydrogen bonds with the polar groups (-OH, -COOH) in the phenolic acids, and the interaction might be too strong to get the analytes eluted down. Therefore, the GnPs was adopted as the appropriate sorbent in this study.

### Sample/sorbent ratio

It is well known that the sample/sorbent ratio is a crucial parameter in MSPDM process that can influences extraction efficiency significantly. A suitable sample/sorbent ratio could improve the effective contact area between adsorbent and sample and facilitate the complete adsorption of sample components on sorbent. In order to achieve the optimal extraction yields, different sample/sorbent ratios were evaluated (4:1, 2:1, 1:1, 2:3 and 1:2), using 24 mg of the sample powder in this study, and the results are shown in Fig. 5B. The peak areas of all the six phenolic acids were found to increase while the sample/sorbent ratio decreased from 4:1 to 1:1. And the highest extraction yields for six analytes were obtained at sample/sorbent ratio of 1:1. The reason for this phenomenon was possibly that along with the increased amount of sorbent, the interaction site on the GnPs augmented and the special interaction between sorbent and analytes was enhanced accordingly, which facilitated a complete adsorption of analytes, and achieved the best extraction performance. However, further increasing the amount of sorbent, i.e. altering the sample/sorbent ratio from 1:1 to 1:2 resulted in reduced extraction yields of all compounds, which was probably due to the overly strong interaction between sorbent and analytes such as π-π stacking interaction, Van der Waals forces and electrostatic interaction which enhanced with the increasing of sorbent amount, that would lead to the difficulty in elution process. Thus, the optimized amount of sorbent was selected at 24 mg in this work.

### The effect of grinding time

In the MSPDM procedure, the grinding time would influence the extraction efficiency of target compounds. Since the sample should be completely dispersed in the sorbent and to form a homogeneous new phase which can promote the good packing of the column and increase the extraction efficiency of the tested compounds. To evaluate the effect of grinding time on the extraction yields of phenolic acids, several dispersion times of 20, 40, 60, 80 and 100 s were investigated with the constant of other experimental conditions. The force applied during the grinding time was kept constant as much as possible. Triplicate experiments of each point with mean values were applied. The results of all the target analytes are showed in Fig. 5C. As can be seen from the graph, the chromatographic peak areas of all the six phenolic acids raised with the grinding time from 20 to 60 s. Possible cause were ascribed to the increased surface contact area between sample and solid phase, as well as extended interaction between adsorbent and sample, both of which are benefit to promote the adsorption of the target analytes into the sorbent. Nevertheless, the peak area responses of all the six analytes decreased a bit and then retained almost unchanged when the grinding time further increased from 60 to 100 s. It is presumed that a completed adsorption and extraction equilibrium of phenolic acids in the adsorption system was attained in 60 s and extending the grinding times, would then lead to an overly strong adsorption ability of the six phenolic acids on the adsorbent due to the tighter combination caused by π-π stacking interaction, Van der Waals forces and electrostatic interaction, which finally increased the difficulty of the desorption procedure. Additionally, the extraction equilibrium may be reversed by further increasing the grinding time over 60 s. Consequently, the grinding time was optimized at 60 s for subsequent experiments.

### Type of elution solvents

The type of elution solvents was another essential parameter in the MSPDM procedure, since determination of target analytes is based on the effectively desorbed components from the MSPDM cartridge. In order to obtain a satisfactory extraction performance for the phenolic acids from the Danshen samples, a variety of elution solutions with different polarities including methanol, ethanol, acetonitrile, acetone, water, methanol-water (80:20, v/v), and methanol-water (60:40, v/v) were investigated. As presented in Fig. 5D, it is clear that water was found to be the most suitable elution solvent compared with the other six elution solvents, presenting largest chromatographic peak areas of all the six tested compounds. As the investigated analytes, i.e. six phenolic acids are highly polar water-soluble compounds which contain -OH and -COOH groups, the highest polarity of water among all elution solvents was supposed for the reason of its best performance. In addition, hydrogen bonds between water and these phenolic acids could further facilitate the dissolution and elution processes. As expected, acetone, due to its weak polarity, eluted the least amount of investigated analytes. Overall, the cheap and environmental friendly reagent water was adopted as the elution solvent in the further work.

### Analytical performances

After optimizing the major influencing parameters in MSPDM procedure evaluation of the proposed MSPDM-UHPLC-ECD method was performed in terms of linearity, precision, the limits of detection (LODs) and quantification (LOQs) under the optimum experimental conditions, i.e. adsorbent of GnPs with sample/sorbent ratio of 1:1, 60 s of grinding time and water as the elution solvent. Triplicate performances were applied, and the results are presented in Table 1, including calibration curve, correlation coefficients (r^2^), precisions, LODs and LOQs. The calibration curves of six phenolic acids were obtained by plotting peak areas versus the concentration of standard mixtures (Fig. S1). Good linearities were observed with the determination  coefficients (r^2^) higher than 0.9991 for all the six compounds in the linear range of 0.5–25 μg/mL. Precision of the developed method was assessed by intra-day and inter-day evaluations on the same sample solution by six replicates of injections in one day and duplicates of injections per day in three consecutive days, respectively. The relative standard deviations (RSDs) of intra-day precision were achieved in the range of 0.80 to 3.59% for peak areas and 0.05 to 0.38% for retention times; while RSD values of inter-day precision were found ranging from 2.41 to 4.57% for peak areas and 0.09 to 0.41% for retention times. These results indicated that the proposed method had good precision and reproducibility. The LODs and LOQs, which were obtained at the signal-to-noise ratios of 3:1 and 10:1 respectively, were calculated to evaluate the sensitivity of the present method, resulting 1.19–4.62 ng/mL and 3.91 to 15.23 ng/mL for LODs and LOQs of all analytes, which indicated sufficient sensitivity for determination of six phenolic acids in real samples.

**Table 1:** Linear Regression Data, Precision, Limits of detection (LODs) and Limits of quantification (LOQs) of the Investigated Compounds

Analyte	Calibration curve	*r* ^2^	Precision (RSD%)				LOD	LOQ
			Intra-day n = 6		Inter-day n = 6			
			Retention time	Peak area	Retention time	Peak area	ng/mL	ng/mL
Sodium danshensu	y = 280.45 x + 58.275	0.9991	0.06	1.11	0.22	3.78	1.94	6.39
Protocatechuic aldehyde	y = 667.14 x + 128.85	0.9992	0.07	0.80	0.15	2.43	1.19	3.91
Rosmarinic acid	y = 170.82 x + 30.748	0.9996	0.07	3.59	0.23	4.57	4.25	14.03
Lithospermic acid	y = 281.12 x + 54.368	0.9991	0.05	1.15	0.19	2.41	3.61	11.93
Salvianolic acid B	y = 152.62 x + 29.499	0.9995	0.38	2.56	0.41	2.56	4.62	15.23
Salvianolic acid A	y = 261.54 x + 52.301	0.9992	0.11	1.24	0.09	4.16	3.70	12.22

### Analysis of samples

**Figure 6:**
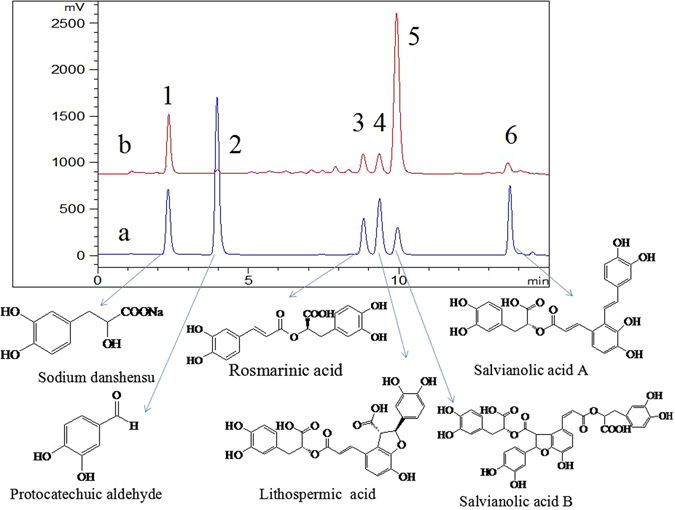
UHPLC-ECD chromatograms of six phenolic acids in standard mixture solution (25 μg/mL) and Danshen tablets pretreated with MSPDM method: a: 25 μg/mL of standard mixtures; b: Danshen tablets sample.

In order to evaluate the applicability of the proposed MSPDM-UHPLC-ECD method in real samples, determination of Danshen tablets under the optimized conditions were used to assess the accuracy and reproducibility of this method. Triplicate experiments were performed. Results are summarized in Table 2, and the UHPLC chromatograms of the standard mixture of six phenolic acids (a) and extracts of Danshen tablet sample (b) are showed in Fig. 6. From the Table 2, the contents of six phenolic acids including sodium danshensu, protocatechuic aldehyde, rosmarinic acid, lithospermic acid, salvianolic acid B and salvianolic acid A in the Danshen tablet sample were 0.81, 0.37, 0.52, 0.34, 6.04 and 0.21 mg/g, respectively. Additionally, the repeatability of the method was investigated by performing three parallel extracts of Danshen tablet sample, the RSDs of the retention time and the peak area were found in the ranges of 1.85 to 3.58% and 3.33 to 5.37%, respectively, indicating the satisfactory reproducibility for this method. Furthermore, recovery test was performed to evaluate the accuracy by using the spiked samples which were added with the standard phenolic acid stock solutions at two different concentration levels (1 and 10 μg/mL). As displayed in Table 2, the mean recoveries of all the six target analytes at two different levels were in the range of 82.34 to 98.34%. Therefore, the proposed method had sufficient accuracy and reproducibility for the extraction and determination of phenolic acids in real samples.

**Table 2:** The Content, Reproducibility and Average Recovery of Samples.

Analyte	Content (mg/g)	Repeatability (RSD%) n = 3		Average recovery	
		Retention time	Peak area	Added (μg/mL)	Recovery%
Sodium danshensu	0.81	2.46	4.09	1	98.34
				10	83.74
Protocatechuic aldehyde	0.37	1.85	3.33	1	95.60
				10	85.31
Rosmarinic acid	0.52	2.32	3.89	1	90.83
				10	86.45
Lithospermic acid	0.34	3.58	5.37	1	89.52
				10	86.37
Salvianolic acid B	6.04	2.67	4.62	1	92.34
				10	89.64
Salvianolic acid A	0.21	1.94	3.85	1	90.66
				10	82.34

### Comparison of the proposed method with previously reported methods

Previous reports of extraction and determination of active compounds in various Danshen samples and its preparations mainly involved the methods of ultrasound assisted extraction (UAE), ionic liquid-based ultrahigh pressure extraction (IL-UPE), shake and sonication extraction, solid-phase extraction (SPE), sonication and ultrasound-assisted ionic liquid-based homogeneous liquid-liquid microextraction (UA IL-based HLLME). The comparison among these methods mentioned above was summarized in Table 3, in terms of sample amount, type and volume of extraction solvent, extraction time, instrumental technique and LODs. As observed from the Table, the proposed MSPDM consumed much less sample amount (24 mg), smaller extraction solvent volume (0.2 mL) and shorter extraction time (1 min) than all the other published methods, without compromising of any sensitivity. In addition, our proposal exhibited a significant advantage of altering toxic organic solvent into eco-friendly water as the solvent for the MSPDM process. Furthermore, the LODs for the MSPDM-UHPLC-ECD method was extremely low compared with other traditional methods which was in the range of 1.19–4.62 ng/mL, indicating the excellent sensitivity of the proposed method. It should be noted that the MSPDM procedure was mild and the heating process was not required, thus the degradation and loss of target analytes could be avoided efficiently, and the equipment used in MSPDM was cheap and easily obtained. In addition, the proposed method was further compared with the SPE method which used graphene nano platelets as the sorbent and the previous report which used miniaturized matrix solid-phase dispersion approach, the data summarized in Table 3 indicated that the two reported method consumed more extraction time and organic solvents. Overall, the established MSPDM-UHPLC-ECD method is a simple, rapid, economic, time-saving, sensitive and eco-friendly method for the analysis of target compositions from real samples.

**Table 3:** Comparison of the proposed method with previously reported methods.

Analyte	Sample (mg)	Sorbent (mg)	Extraction solvent^c^ (mL)	Extraction time (min)	Extraction method^a^	Instrumental technique^b^	LOD (ng/ mL)	References
Tanshinone IIA, tanshinone I, cryptotanshinone, dihydrotanshinone, salvianolic acid B	Danshen and its preparations, 500 mg	—	70% methanol, 20 mL	20	UAE	LC-DAD	20–140	36
Protocatechuic acid, protocatechuic aldehyde, caffeic acid, ferulic acid, isoferulic acid, rosmarinic acid, salvianolic acid B, salvianolic acid A, dihydrotanshinone I, przewalskin, cryptotanshinone, tanshinone I, tanshinone IIA	*Salvia Miltiorrhiza*, 500 mg	—	Methanol, 25 mL	45	UAE	UPLC-PDA	10–350	45
Danshensu, protocatechuic aldehyde, notoginsenoside R1, ginsenoside Rg1, salvianolic acid B, ginsenoside Rb1, cryptotanshinone, tanshinone IIA	Fufang Danshen preparations, 500 mg	—	70% methanol, 25 mL	30	UAE	HPLC-DAD-ELSD	40–1290	46
Tanshinone I, tanshinone IIA, cryptotanshinone	*Salvia Miltiorrhiza* Bunge, 50 mg	—	50 mM C_16_mimBr aqueous solution, 4 mL	30	UAE	HPLC-DAD	10–50	47
Dihydrotanshinone, cryptotanshinone, tanshinone I, tanshinone IIA, miltirone	*Salvia Miltiorrhiza Bunge*, 500 mg	—	[C_8_MIM][PF_6_] in ethanol, 10 mL	2	IL-UPE	HPLC	8.2–23.1	37
Danshensu, protocatechuic aldehyde, salvianolic acid B, dihydrotanshinone I, cryptotanshinone, tanshinone I, tanshinoneIIA	*Radix salviaemiltiorrhizae*, 500 mg	—	70% methanol in water (v/v), 40 mL	60	UAE	HPLC-UV	6.9–48.7	48
Danshensu, protocatechuic aldehyde, salvianolic acid B, cryptotanshinone, tanshinone I, tanshinone IIA	Danshen, 30 mg	—	Methanol-Water (80:20, v/v), 4.5 mL	45	Shake and sonication	HPLC-PAD	20–90	38
Cryptotanshinone, tanshinone I, tanshinone IIA	*Salvia Miltiorrhiza Bunge*, 500 mg	Ionic liquid-modified silica	Methanol, 50 mL and methanol-acetate acid (90:10,v/v), 2 mL	240	SPE	HPLC-UV	76–93	39
Salvianolicacid B, lithospermic acid	Danshen tablets, 250 mg	—	Electrolyte solution, 20 mL	15	Sonication	MEKC-UV	100–500	40
Dihydrotanshinone, tanshinone I, cryptotanshinone, tanshinone IIA	*Salvia Miltiorrhiza* Bge. root., 10 mg	—	water, 5 mL and [C_8_MIM][BF_4_], 0.14 mL	11	UA IL-based HLLME	HPLC-UV	52–93	41
3-Chlorophenol, 4-chlorophenol, 2,4-dichlorophenols	Water, 50 mL	Graphene nanoplatelets (30 mg)	Methanol, 9 mL; water, 9 mL; 10% (v/v) methanol, 1 mL and alkaline methanol, 2 mL	50	SPE	UV-vis spectrophotometry	—	18
Fenpropathrin, cyhalothrin, fenvalerate	Soil, 100 mg	Silica (300 mg)	Acetone, 3 mL; tetrachloroethylene, 50 μL; water, 5 mL	12	MSPD-UADLLME	GC-ECD	0.45–1.13 ng/g	49
Sodium danshensu, protocatechuic aldehyde, rosmarinic acid, lithospermic acid, salvianolic acid B, salvianolic acid A	Danshen tablet, 24 mg	Graphene nanoplatelets (24 mg)	Water, 0.2 mL	1	MSPDM	UHPLC-ECD	1.19–4.62	This method

^a^UAE, ultrasound assisted extraction; IL-UPE, ionic liquid-based ultrahigh pressure extraction; SPE, solid-phase extraction; UA IL-based HLLME, sonication and ultrasound-assisted ionic liquid-based homogeneous liquid-liquid microextraction; MSPD-UADLLME, matrix solid-phase dispersion combined with ultrasound-assisted dispersive liquid-liquid microextraction; MSPDM, matrix solid phase dispersion microextraction.

^b^LC-DAD, liquid chromatography-diode array detection; UPLC-PDA, ultraperformance liquid chromatography-photodiode array; HPLC-DAD-ELSD, high performance liquid chromatography coupled with diode array and evaporative light scattering detectors; HPLC-DAD, high-performance liquid chromatography-diode array detection; HPLC, high-performance liquid chromatography; HPLC-UV, high-performance liquid chromatography-ultraviolet detector; HPLC-PAD, high-performance liquid chromatography-photodiode array detector; MEKC-UV, Micellar electrokinetic chromatography-ultraviolet detector; GC-ECD, gas chromatography coupled with electrochemical detection; UHPLC-ECD, ultrahigh performance liquid chromatography coupled with electrochemical detection

^c^C_16_mimBr,1-methyl-3-hexadecylimidazolium bromide; [C_8_MIM][PF_6_],1-octyl-3-methylimidazolium hexafluorophosphate; [C_8_MIM][BF_4_], 1-octyl-3-methylimidazolium tetrafluoroborate

In this study, a novel, simple and effective GnPs based MSPDM combined with UHPLC-ECD method was developed and validated for the simultaneous extraction and determination of six phenolic acids in a plant preparation, Danshen Tablets. In this proposed MSPDM procedure, the GnPs were used as the sorbent and the water was adopted as the elution solvent which was consistent with the requirements of green chemistry. Compared with other reported approaches, this method provided advantages as the lower cost of sample and analysis time, inorganic solvent as the elution solvent, and high sensitivity. Furthermore, the results of the validation of the method demonstrated that the proposed MSPDM-UHPLC-ECD method had satisfactory accuracy, precision, and sensitivity for the extraction and determination of phenolic acids in Danshen tablets and other plant samples.

## Methods

### Reagents and materials

The GnPs, graphene and graphene oxide (powder) were obtained from Sinopharm Chemical Reagent Co., Ltd. (Shanghai, China). Graphene oxide (sheet) was provided by Nanjing XFNano Material Tech Co., Ltd. (Nanjing, China). The alumina (Al_2_O_3_) was purchased from ANPEL Scientific Instrument Co., Ltd. (Shanghai, China). C_18_ was supplied by Shanghai Chengya Chemical Co., Ltd. (Shanghai, China). The single-walled carbon nanotube (SWNT), HPLC grade sodium dihydrogen phosphate and phosphoric acid were obtained from Sigma-Aldrich Shanghai Trading Co., Ltd. (Shanghai, China). Chromatographic grade methanol and acetonitrile were purchased from Tedia Company Inc. (Fairfield, US). Analytically pure ethanol was provided by Hangzhou Chemical Reagent Co., Ltd. (Hangzhou, China). Acetone (analytical grade) was obtained from Quzhou Juhua Reagent Co. Ltd (Quzhou, China). Purified water was purchased from Hangzhou Wahaha Group Co., Ltd. (Hangzhou, China). The 0.2 μm disposable nylon membranes (diameter: 50 mm) were supplied by Jinteng Laboratory Equipment Co., Ltd. (Tianjin, China). Six standard phenolic acids including sodium danshensu, protocatechuic aldehyde, rosmarinic acid, lithospermic acid, salvianolic acid B and salvianolic acid A were provided by Shanghai Winherb Medical Technology Co., Ltd. (Shanghai, China), with the purity ≥98%. The standard stock solutions were prepared by dissolving each appropriate amount of standard in methanol at a concentration of 200 μg/mL, and the working solutions were obtained by diluting the stock solutions with methanol before use. The Danshen tablets were acquired from Hangzhou local drugstore (Hangzhou, China).

### MSPDM procedure

Several parameters including the type of sorbent, sample/sorbent ratio, grinding time, type of elution solvents, which affected the extraction performance of phenolic acids from Danshen tablets by MSPDM method were systematically investigated and optimized through the single element experiment. Each parameter was experimented in triplicate, and the mean results were applied for evaluation. The extraction process was as follows:

Danshen tablets were comminuted and sieved through a no. 60 mesh. Then, a certain amount of the Danshen tablets powder and sorbent (GnPs, graphene, graphene oxide (sheet), graphene oxide (powder), Al_2_O_3_, C18 or SWNT) were accurately weighed at the sample/sorbent ratio of 4:1, 2:1, 1:1, 2:3 and 1:2 and placed into an agate mortar and blended using a pestle for 20–100 s to obtain a homogeneous mixture. The mixture was introduced into a 1 mL SPE cartridge with a sieve plate at the bottom. A second sieve plate was placed on the top of the blend and slightly compressed with a syringe plunger. Next, 0.2 mL of elution solvent (methanol, ethanol, acetonitrile, acetone, water, methanol-water (80:20, v/v), and methanol-water (60:40, v/v)) was loaded to elute the analytes by using an oil pump. The eluate was collected in a 2 mL Eppendorf tube and diluted 10 times with elution solvent. At last, the solution was centrifuged for 5 min at 13,000 rpm and 2 μL was injected into the UHPLC-ECD system for analysis.

### Instrumental conditions

The analyses of phenolic acids were performed on an Agilent 1290 system (Santa Clara, CA) equipped with an Antec SDC ECD (Antec, Netherlands). The separation was carried out on an Agilent SB-C18 column (1.8 μm, 4.6 mm i.d. × 50 mm) which was maintained at 35 °C. The flow rate was set at 0.5 mL/min, and the injection volume was 2 μL. The mobile phase made up of 25 mM sodium dihydrogen phosphate buffer with 5% (v/v) methanol (pH 3.5) (A) and 25 mM sodium dihydrogen phosphate buffer with 80% (v/v) methanol (pH 3.5) (B). The pH of the mobile phase was adjusted by using phosphoric acid. The gradient of mobile phase was as follows: 0–2 min, 20–42% B; 2–3 min, 42–43% B; 3–4 min, 43–44% B; 4–5 min, 44–45% B; 5–9 min, 45–45% B; 9–10 min, 45–50% B; 10–12 min, 50–100% B; 12–15 min, 100–100% B. The electrochemical cell was a SenCell of a confined wall-jet design. The detection potential of ECD was E_cell_ = +0.70 V and the detector was set at 500 nA range, and the Ag/AgCl reference electrode was used. The morphologies and microstructures of GnPs and GnPs-Danshen tablets were characterized by a SEM (HT7700, Hitachi, Tokyo, Japan) and TEM with a Supra55 microscope (Zeiss, Oberkochen, Germany) with an acceleration voltage of 100 kV. The infrared spectra of GnPs, Danshen tablets and GnPs-Danshen tablets were determined by a fourier transform infrared (FT-IR) spectra which were recorded on a Thermo Scientific Nicolet iS5 spectrometer (Madison, USA). The sample used for FT-IR scan was prepared by grinding with KBr powder and then pressed into a pellet. Ver551B AFM was used to investigate the thicknesses of the GnPs, graphene, graphene oxide (sheet) and graphene oxide (powder) with silicon cantilevers “NSC15/AIBS” (μmash; NanoNavi, Japan).

## Electronic supplementary material

Supplementary material
