# Supplementary material for: Graphene nanoplatelets based matrix solid-phase dispersion microextraction for phenolic acids by ultrahigh performance liquid chromatography with electrochemical detection
Source: Sci Rep. 2017 Aug 8;7:7496. doi: 10.1038/s41598-017-07840-2 (PMC5548748; doi:10.1038/s41598-017-07840-2)

**Graphene nanoplatelets based** **matrix solid-phase dispersion microextraction for phenolic acids by ultrahigh performance liquid chromatography with electrochemical detection**

Li-Qing Peng^1^, Ling Yi^2^, Qiu-Cheng Yang^1^, Jun Cao^1*^, Li-Jing Du^1^ & Qi-Dong, Zhang^1^

^1^*College of Material Chemistry and Chemical Engineering, Hangzhou Normal University, Hangzhou 310036, China*

^2^*Drug Clinical Trial Institution, The First Affiliated Hospital of Soochow University, Suzhou 215006, P. R. China*

* Corresponding author: Dr. Jun Cao

**E-mail:** caojun91@163.com

**Tel.**: +86 571 2886 7909

**Fax:** +86 571 2886 7909

**FIGURE CAPTIONS**

**Fig. S1.** The calibration curves of six phenolic acids obtained by plotting chromatographic peak areas versus the concentration of standard mixtures.

**Figure S1**


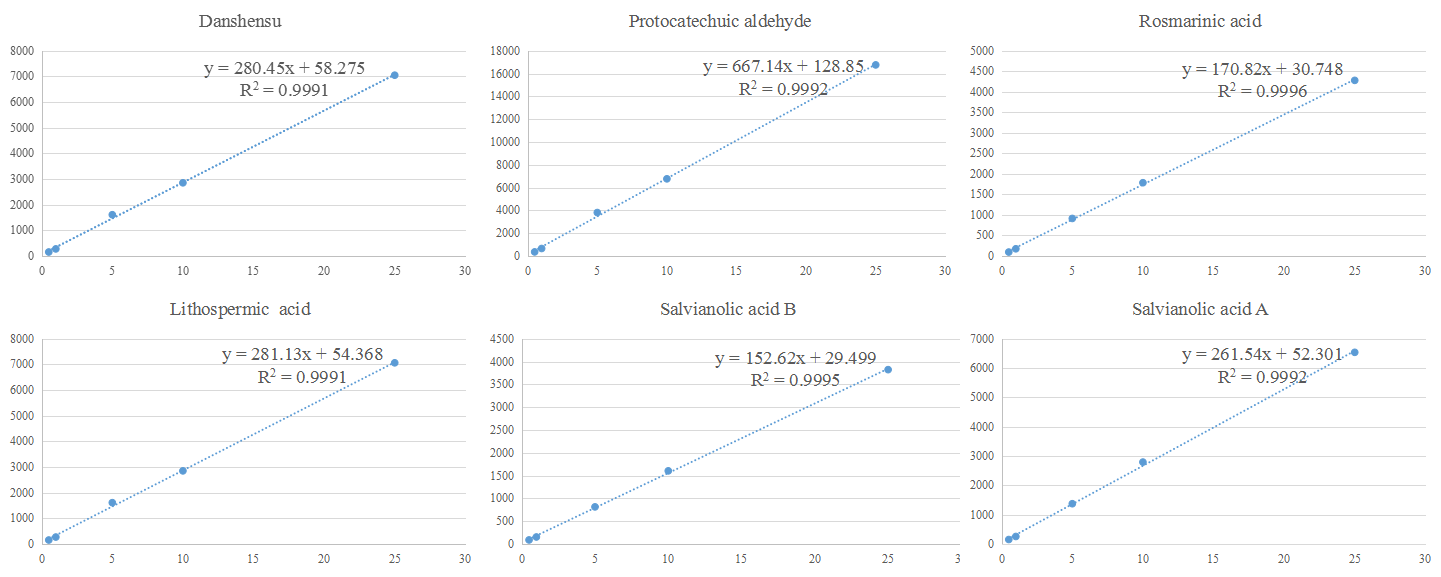

Supplement: Supplementary file 1 — Supplementary material [file 41598_2017_7840_MOESM1_ESM.docx]
